# Can community health centers improve the self-rated health of migrants? Evidence from China

**DOI:** 10.3389/fpubh.2022.986201

**Published:** 2022-09-23

**Authors:** Ai-Lin Mao, Yu-Kun Tian, Ya-Nan Li

**Affiliations:** School of Labor Economics, Capital University of Economics and Business, Beijing, China

**Keywords:** community health center, self-rated health (SRH), migrant, China migrants dynamic survey, China

## Abstract

**Background:**

Due to the “epidemiological paradox,” migrants face the risk of health attrition during their migration. Meanwhile, institutional constraints cause a health gap between migrants and non-migrants. To narrow this gap and maintain equity, scholars have studied the role and impact mechanism of medical insurance participation in improving the health of migrants. However, due to the provision of China's basic medical insurance system, the proportion of migrants participating in employee medical insurance is still relatively low, while the community health center (CHC) is a more accessible medical resource for this group. Therefore, this study attempts to explore the impact of CHCs on the self-rated health (SRH) of migrants and identify the factors and mechanisms associated with such an impact. This study addresses the hypotheses whether (a) CHCs can significantly improve the SRH of migrants in China and (b) CHCs improve the SRH of migrants by promoting both their health knowledge and health behavior.

**Methods:**

Data was obtained from the 2017 China Migrants Dynamic Survey (CMDS). From the survey, 127,687 migrants were identified, and a series of logit regressions were conducted to explore the correlation between CHCs and the SRH of migrants. Propensity score matching (PSM) logit was also used for the robustness tests.

**Results:**

Logit estimations revealed that CHC is positively related to the SRH of migrants (OR = 1.095, *p* < 0.001). Compared to others, males (OR = 1.156, *p* < 0.001), younger people with higher education (OR = 1.027, *p* < 0.001), more stable employment (OR = 1.544, *p* < 0.001), and people with a lower proportion of elderly (> 65 years) household family members (OR = 0.842, *p* < 0.001) tended to have better SRH. The results also showed that the impact of CHCs on migrants' SRH varied by gender, age, and income (*p* < 0.001). A possible mechanism is that CHCs can improve migrants' SRH by promoting both their health knowledge and health behaviors.

**Conclusion:**

Programs that strengthen health knowledge and policies to enhance access to healthcare could be prioritized to improve the SRH of migrants in China.

## Introduction

Since China's reform at the end of the 1970s, with urbanization and economic growth, a large number of migrant workers in China have entered cities for employment opportunities (1). The number of migrants has grown dramatically since the 1980s, especially from the 1990s to 2010. It has increased from approximately 2 million in 1983 to 221 million in 2010 and 376 million in 2020. Based on the latest national census of China, this group still occupies an important position with a scale of 26% of the total population (2). However, many migrant workers can only engage in labor-intensive jobs with longer working hours and lower pay, partly due to their lower education or lack of skills training (3). Previous studies have shown that poor working conditions may cause health issues (4). Empirical research has proven that the above conclusions are applicable to migrants in China. A regional survey conducted in China showed that the smoking rate among migrant males was 48.12%, which was higher than that of urban adult men (39.60%), and the drinking rate also showed similar results (62.40% among migrant males and 11.7% among urban adult males), which resulted in overweight, obesity, hypertension, and dyslipidemia among migrants (5). According to the results of another study, young and middle-aged Chinese female migrants had a higher rate of tobacco and alcohol use than the average level of Chinese women, which is significantly associated with an increased health risk of breast hyperplasia in this group (6). Meanwhile, due to institutional constraints, it is difficult for migrants to obtain urban household registration and social welfare binding with it. Therefore, the health dilemma faced by migrants in China is that they face relatively higher health risks and are unable to obtain more medical security resources because of institutional restrictions (7). This makes migrant health a research topic worthy of attention.

The “epidemiological paradox” confirmed that the health status of migrants deteriorated with the increase of migration years (8). Studies have confirmed that these effects also exist in China (9). Leaving this question unaddressed will create health disparities between migrant and non-migrant groups, and lead to social inequity. Current studies have been trying to address this problem based on the “Anderson model” and have found that medical insurance may help migrants maintain health by improving their health knowledge and behavior.

However, these studies did not consider that migrants also have institutional limitations in participating in medical insurance. Meanwhile, as a primary medical service institution, the medical resources provided by the community health centers (CHCs) are not limited to the migrants. Therefore, this paper will study whether CHCs improve the health of migrants in China according to the “Anderson model”, in order to help the migrants alleviating the deterioration of health and maintain social equity.

## Literature review

### Self-rated health

Self-rated health (SRH), also known as self-assessment and self-reported health, is an individual's subjective evaluation of their current health status (10). Early research showed that aging people's SRH was a better predictor of seven-year survival than medical records (11). This conclusion is also applicable to the middle-aged group, which showed that for the age group of 45-64 years old, the mortality rate of those with poor SRH was twice as high as that of self-reported good health (12). More recent studies have shown that SRH is a reliable predictor of morbidity and mortality (13) and is associated with a variety of chronic diseases, such as dementia, diabetes, congestive heart failure, cerebrovascular disease, and coronary artery disease (14).

SRH is distinct from other indicators that have been traditionally used to study the health status of migrants, such as medication-seeking behaviors and chronic diseases (5), which tend to focus on one specific aspect of health. SRH is seen as the evaluation of one's health as a whole, and it is generally believed that, although SRH cannot be exactly consistent with objective health, there are still studies that have confirmed that there is a significant positive correlation between SRH and objective health (15). The SRH is a relatively stable and valid health status indicator that can be used in cohort studies and population health monitoring (16).

With the rapid increase in migrants in China and the fact that they are facing intense health risks, it is important to understand the factors that may contribute to their health. Therefore, SRH was used in this study as an indicator of migrants' health.

### Health of migrants: Theory

The “healthy immigrant effect” (HIE) assumes that because the behavior of mobility itself requires good physical and mental health conditions, immigrants should be healthier than those who have not migrated or moved (17). Correspondingly, the “salmon bias” has arisen, which refers to the phenomenon that immigrants return to their place of origin after retirement, unemployment, or serious illness. The combination of these two effects leads to an “epidemiological paradox” that migrants' health will experience attrition, and finally, the health advantage of HIE disappears (18). Some Chinese scholars have also introduced this paradox to study the problem of migrant workers' health, and generally agree that the health status of migrants will gradually deteriorate with the accumulation of migration time, suggesting that this effect also exists among Chinese migrants (19). This “health attrition” is an important reason for the health gap between migrants and urban residents in China (9), in which, the mechanism might be related to medical access (20). Empirical studies in China have shown that migrants face more health risks (21) and have less utilization of medical services (22). A study of Mexican and Middle Eastern immigrants shows that increasing medical service utilization can effectively improve immigrants' health (23), which implies that medical accessibility is a factor in reducing this “epidemiological paradox”. However, there is still a lack of sufficient empirical evidence regarding whether this conclusion is applicable to migrants in China.

The equality of opportunity theory suggests that a person's advantage caused by an uncontrollable factor which is called “circumstance” is unreasonable that may lead to inequality of opportunity (24). Researchers later introduced this theory to health economics; hence, the “circumstance” factors that cause inequality in individual health might include the household registration and socioeconomic status division (25). Scholars have investigated ways to reduce this inequality (26). As stated above, the migrants in China face “epidemiological paradox,” of which quo widen the health gap between the migrant and non-migrants and cause inequity. Since it takes time to adjust the system, discussing whether the existing medical resources available to migrants can help maintain their health, thereby alleviating their health attrition.

### Health of migrants: Evidence

Although there is very little research exploring these hypotheses, few studies have yielded results and were mostly conducted with non-representative samples of the population. Based on the “Anderson model,” individual and contextual characteristics may correlate with health behavior and ultimately result in health outcomes. Several studies have been conducted based on this theoretical framework. For instance, research has shown that social support, especially belongingness and integration, play a key role in promoting the general mental health of migrants (27). The main channel of the network effect is boosting confidence and reducing anxiety (28). This type of research mostly focuses on the influence of informal social support on the mental health of migrants (29) from a psychological perspective but lacks discussions on formal social support.

Another research field is the impact of medical insurance on migrants' health. The rationale is basically following the “Anderson model”, which states that participating in medical insurance can increase the medical-seeking behavior of migrants, thereby improving their health (30, 31). Studies have proven that social medical insurance significantly improves the health of insurance beneficiaries (32), especially because of the disadvantages of increasing the use of medical services (33). Some researchers have examined the health promotion effect of various medical insurance schemes in China separately and obtained similar results (34). However, a comparative study showed that urban employee basic medical insurance (UEBMI) for urban employees and urban resident basic medical insurance (URBMI) for urban citizens increased the likelihood of migrant workers having better SRH than the new cooperative medical scheme (NCMS) for rural citizens (35). This suggests that due to the non-portability of rural medical insurance schemes, their effectiveness in improving migrant workers' health status is limited (36).

In summary, although prior research indicates that informal support and medical insurance can improve migrants' health, it remains unclear whether other factors, especially other forms of formal social support that migrants can utilize, may result in similar consequences. Based on the “Anderson model,” CHCs could be a contextual factor that plays a substantial role in local communities and the health of disadvantages (37). It is also the main form of primary care provision in China, as it is community-based and does not have access limitations on migrants (38). In addition to basic disease diagnosis and treatment, these services also include prevention, health care, rehabilitation, and health education. Since the launch of the “new medical reform” in 2009, China has begun to attach importance to the construction of primary medical institutions. For this reason, the number of CHCs has increased from 8,211 in 2002 to 35,000 in 2021, basically achieving the goal that CHCs covering every district. At present, the coverage rate of CHC in all regions of China is over 50% (56.5% in eastern regions, 57.2% in central regions, and 62.5% in western regions) (39).

Therefore, as a potential contextual characteristic that may affect health, the advantage of CHCs over medical insurance for migrants in China currently lies in fewer institutional restrictions. However, whether an increase in CHCs results in improved wellbeing among migrants remains critically unexplored, which might have contributed to the mixed findings in the literature. Using a nationally representative sample of migrants, the main purpose of this study was (a) to investigate the potential impact of CHCs on SRH, (b) to identify heterogeneity, and (c) to determine the mechanism by which CHCs improve migrants' SRH.

Thus, this study seeks to build on previous research to explore the following hypotheses:

**Hypothesis 1**: CHCs can significantly improve the SRH of the migrants in China.
**Hypothesis 2**: CHCs improve migrants' SRH by promoting both their health knowledge and health behavior.

## Methods

### Data source

The data adopted in this paper was from the “2017 China Migrants Dynamic Survey (CMDS),” which is an annual large-scale national sampling survey of the migrants conducted by the National Health Commission since 2009. The survey uses proportional to population size (PPS) to select the inflow places with relatively concentrated migrants from 31 provinces (autonomous regions and cities) and Xinjiang Production and Construction Corps. Inflow people aged 15 and above who live in the sample points of the inflow places for 1 month or more were sampled. The main survey contents included basic demographic characteristics, employment, income and expenditure, health status, and basic public services of the migrants, so the survey reflected the basic situation and public health services of migrants in this group. Therefore, this study uses only 2017 CMDS data, since CMDS annual survey contains inconsistent questions. The total sample size for this survey was approximately 170,000 individuals. After screening and merging variables, the effective sample size was determined to be 127,687.

### Variables

#### Dependent variables

Table 1 presents the definitions and assignments for each variable. This study investigates whether CHCs can improve migrants' SRH. Therefore, SRH was the main dependent variable. The relevant survey question was “How is your health status?”, with four possible types: healthy, basically healthy, unhealthy but able to take care of oneself, and unable to take care of oneself. Among them, the “healthy” response is coded as “1,” and the rest coded as “0”.

**Table 1:** Descriptive statistics and assignment of the variables.

**Variable's name**	**Mean**	**SD**	**Min**	**Max**	**Assignment**	**Percent (%)**
**Health status**						
Self-rated health	0.83	0.38	0	1	Healthy = 1	82.80
					Other = 0	17.20
Sickness or not	0.50	0.50	0	1	Sickness = 1	49.74
					Not sick = 0	50.26
**CHC**						
Is there a CHC	0.58	0.49	0	1	Yes = 1	57.89
					No = 0	42.11
**Individual characteristics**						
Age	36.49	10.95	15	96		
Gender	0.51	0.50	0	1	Male = 1	51.28
					Female = 0	48.72
Nationality	0.93	0.26	0	1	Han nationality = 1	92.73
					Other = 0	7.27
Year of education	10.33	3.37	0	19		
Marital status	0.82	0.39	0	1	Married = 1	81.87
					Unmarried = 0	18.13
Migration time	7.26	5.86	1	27		
**Employment characteristics**						
Employment status	0.83	0.38	0	1	Employed = 1	83.06
					Unemployed = 0	16.94
**Characteristics of family**						
Proportion of people aged 0-6 in the family	0.11	0.16	0	0.67		
Proportion of people aged 65 and over in the family	0.02	0.11	0	1		
per capita household monthly income	2,593	1,959	200	13,333		
**Regional characteristics (city level)** ^**1**^						
Unemployment rate	0.02	0.01	0.002	0.124		
Per capita GDP	92,997	39,905	12,656	191,942		

The CMDS data does not include regional economic indices, for the purpose of this study, we include these data from “China Urban Statistical Yearbook (2017)” and match these indices to samples by region (at city level).

#### Independent variables

In previous studies, some scholars have investigated the impact of informal social support on migrants' wellbeing. Other scholars have investigated the impact of medical insurance on migrants' health. However, there are other factors that contribute to maintaining the health of migrants, thereby slowing their health loss. Meanwhile, studies in the US have shown that CHCs have improved some aspects of the health of disadvantaged people. China has attached importance to the construction of CHCs in the past decade. Given this background, this study examines whether CHC's formal support can also improve the health of migrants in China. Therefore, this study used CHCs as the main independent variable, which was measured by the question “Is there a CHC in your community?”, and “yes” coded as “1”, “no” coded as “0”.

#### Control variables

Based on the “Anderson model” and the aim of this study, we chose four types of variables as covariates (40): the first category is the individual characteristics of the migrants, including age, gender, nationality, year of education, marital status, and migration time; the second category is employment characteristics, including employment status (employed or unemployed); the third category is family characteristics, including variables reflecting family demographics and economic status, which were based on the proportion of population of aged 0–6 and ≥65 in the family per capita household income. This study also controls for regional variables at the city level, including unemployment rate and per capita GDP.

## Results

### Descriptive statistics

Table 1 shows that among the total sample (*n* = 127, 687), 82.8% considered themselves healthy, while 49.74% reported experiencing illness or physical discomfort in the previous year. This shows that migrants' subjective perceptions of health are inconsistent with their objective situations. This further indicates that migrants have lower requirements for health and a higher tolerance for diseases, which may lead them to be reluctant to receive medical treatment. Table 1 also shows that 57.89% of the sampled migrants live in a community with CHCs, which reflects the strength of China's construction of CHCs. This is also consistent with our premise that CHCs are a crucial form of social support in China.

Table 1 shows that the migrants were relatively young, with an average age of 36.49. According to the maximum and minimum ages, there was a flow of seniors and juveniles whose health risks are relatively higher. In addition, the average number of years of education in the sample is 10.33, of which the minimum education level is illiterate. Table 1 shows that most of the participants were of Han nationality, with a ratio of 92.73%. The number of males exceeded that of females. More than 80% of respondents were married. The average migration year was 7.26. In terms of employment characteristics, approximately 83.06% of these respondents were employed; thus, it can be inferred that employment remains the primary reason for mobility in this group. The average monthly household earnings are about 2,593 renminbi (RMB) on average, with a minimum of 200 RMB and a maximum of 13,333 RMB. Even when considering regional income differences and the existence of unemployed household members, this result reflects the low-income situation of migrants. Regarding the demographic structure of migrant families, the average proportion of children aged 0–6 was 11%, followed by 2% of elderly individuals over 65. For regional characteristics, the average unemployment rate and per capita GDP were 2% and 92,997 RMB, respectively.

### Univariate test

The chi-squared test was used to analyze categorical variables. Table 2 shows that there were differences in SRH among different migrants (*p* < 0.001). The migrants who were male (83.44%), unmarried (88.14%), and employed (85.07%) had better SRH. Meanwhile, migrants who were of Han nationality (82.85%) showed better SRH (p <0.1).

**Table 2:** Univariate test (1).

**Variables**		**Self-rated health** ***N*** **(%)**		**χ^2^**	***p***
		**Other**	**Healthy**		
Gender	Female	11,120 (17.87)	51,090 (82.13)	39.003	<0.001
	Male	10,840 (16.56)	54,637 (83.44)		
Nationality	Other	1,654 (17.83)	7,623 (82.17)	2.795	<0.1
	Han nationality	20,306 (17.15)	98,104 (82.85)		
Marital status	Unmarried	2,747 (11.86)	20,407 (88.14)	565.12	<0.001
	Married	19,213 (18.38)	85,320 (81.62)		
Employment status	Unemployed	6,127 (28.33)	15,503 (71.67)	2.3e + 03	<0.001
	Employed	15,833 (14.93)	90,224 (85.07)		

An independent samples *t*-test was used to analyze continuous variables, and the results showed that older migrants had worse SRH (42.99 > 35.14 years old). Longer years of education (10.55 > 9.24) and the proportion of family members aged 0–6 years in a household (0.11 > 0.08) are positively correlated with migrants' SRH, while the proportion of family members aged above 65 (0.01 <0.06) and longer years of migration (7.00 <8.54) show the opposite correlation. Per capita income was positively correlated with SRH (2670.55 > 2220.84). Migrants may enjoy better SRH, with a higher per capita GDP (*p* < 0.001) (Table 3).

**Table 3:** Univariate test (2).

**Variables**	**Self-rated health**		**Mean difference**	***t***	***p***
	**Other**	**Healthy**			
Age	42.99	35.14	7.86	100.55	<0.001
Year of education	9.24	10.55	−1.31	−53.06	<0.001
Migration time	8.54	7.00	1.54	35.71	<0.001
Proportion of population aged 0–6 in the family	0.08	0.11	−0.03	−27.45	<0.001
Proportion of population aged 65 and over in family	0.06	0.01	0.05	56.94	<0.001
Per capita household monthly income	2,220.84	2,670.55	−449.71	−31.07	<0.001
Unemployment rate	0.02	0.02	0.00	17.90	<0.001
Per capita GDP	89,512.52	93,720.67	−4,208.15	−14.23	<0.001

### Model

To better understand the impact of CHCs in China on migrants' SRH, we incorporated factors that have been tested by the univariate test into the regression model. Since the dependent variable “self-rated health” and the independent variable “is there a CHC in the community” are both binary, which can be divided into either “healthy/unhealthy” or “yes/no”. Thus, we used a binary logistic regression model constructed as follows:


(1) $$Health_{i}^{*}\;=\;\alpha_{i}\;+\;\beta_{i}CHC_{i}\;+\;\gamma_{i}Z_{i}\;+\;P_{i}\;+\;\varepsilon_{i}$$

Among them, $Health_{i}^{*}$ is the dependent variable, *CHC*_*i*_ is the main independent variable, *Z*_*i*_ is the control variable where the per capita household monthly income and per capita gross domestic product (GDP) are in logarithmic form, *P*_*i*_ is the provincial dummy variable, and ε_*i*_ is the error term.

### Benchmark regression

The variables were submitted to Stata 16.0 and binary logistic regression models were established. The regression results are shown in Table 4, which reflect the impact of CHCs on SRH. For Table 4, Model 1 is the benchmark model that only considers individual characteristics control variables; Model 2 considers both individual characteristics and employment characteristics control variables; Model 3 adds family characteristics control variables based on Model 2; and Model 4 includes all the control variables.

**Table 4:** Logistic regression results of CHCs on SRH of the migrants in China (*N* = 127,687).

**Variables**	**Model 1**		**Model 2**		**Model 3**		**Model 4**	
	**B**	**Exp (B)**	**B**	**Exp (B)**	**B**	**Exp (B)**	**B**	**Exp (B)**
CHC	0.103***	1.108	0.105***	1.110	0.099***	1.104	0.090***	1.095
Age	−0.059***	0.943	−0.055***	0.946	−0.054***	0.947	−0.055***	0.947
**Gender (female)**								
Male	0.242***	1.274	0.142***	1.152	0.145***	1.156	0.145***	1.156
**Nationality (others)**								
Han nationality	0.157***	1.170	0.144***	1.155	0.130***	1.139	0.126***	1.134
Year of education	0.037***	1.037	0.038***	1.038	0.027***	1.027	0.027***	1.027
**Marital status (unmarried)**								
Married	0.103***	1.108	0.097***	1.102	0.146***	1.157	0.148***	1.160
Migration Time	−0.012***	0.988	−0.014***	0.986	−0.013***	0.987	−0.013***	0.987
**Employment status (unemployed)**								
Employed			0.478***	1.613	0.437***	1.548	0.435***	1.544
Proportion of people aged 0–6 in the family					0.006	1.006	0.015	1.015
Proportion of people aged 65 and over in the family					−0.170***	0.843	−0.172***	0.842
Log of per capita household income					0.212***	1.236	0.215***	1.240
Unemployment rate							−6.999***	0.001
Per capita GDP^a^							−2.336***	0.097
Square per capita GDP							0.100***	1.105
*N*	127,687	127,687	127,687	127,687	127,687	127,687	127,687	127,687
pseudo *R*-sq	0.101	0.101	0.105	0.105	0.107	0.107	0.108	0.108

^a^Both of the per capita GDP and square of per capita GDP have been taken logarithm.

***, **, and *represent significance at the 1, 5, and 10% levels, respectively.

#### Effects of CHCs on self-rated health

In Table 4, Model 1 shows that when considering individual characteristics, CHCs have a significant positive impact on SRH. Meanwhile, Model 2 and Model 3 considers employment variables and family characteristics based on Model 1 and finds that these migration and family factors also have great influences on SRH. Finally, Model 4 considers all individual, employment, migration and region characteristics, which reflects the influence of CHCs on the SRH of the migrants at the destination after controlling for all four categories of variables. The statistical results show that having CHC within a community has a significant positive impact on the SRH of the migrants in the destination areas at a significance level of 0.001. Exp (B) of CHC rate is 1.095, which means comparing with those living in a community without CHCs, the odds of having better SRH increase is by a factor of 1.095. Therefore, hypothesis 1 has been tested.

#### Effects of control variables on self-rated health

In terms of individual characteristics except age, all other individual characteristics had significant positive effects on SRH. The SRH of migrants increases 0.947 times as their age decreases by one unit. This implies that younger migrants tend to have better SRH. Male migrants tend to be more confident in their health with an odds ratio of 1.156 for self-rated “healthy” compared to women. Similarly, people of Han nationality reported better SRH than minorities. In addition, the years of education indicate that migrants with longer education experience would have better SRH. The results show that for each additional year of education, the odds ratio of self-rated as “healthy” increased by a factor of 1.027. Compared with single people, people who stay married show better SRH, as Exp (B) is 1.160. The result of migration time confirms the “epidemiological paradox” that for each additional migration year, the odds ratio of self-rated as “unhealthy” increases by a factor of 0.987.

Regarding employment characteristics, a significant positive correlation was found between employment status and SRH. Compared to people who are unemployed, the odds ratio of self-rated as “healthy” will increase by 1.544 if people are employed.

For family characteristics, there was a significant negative correlation between the proportion of elderly household members and SRH at the destination, whereas the correlation between the proportion of household children aged 0–6 and SRH was not significant. The odds ratios of the proportion of household children aged 0–6 and the proportion of household senior family members were 1.015 and 0.842, respectively. Regarding the per capita household monthly income, the lower the income is, the less likely the migrants to consider themselves “healthy”.

In terms of regional characteristics, the unemployment rate was negatively correlated with SRH, with an odds ratio of 0.001. The correlation between SRH and per capita GDP shows a “U shape” with GDP and SRH negatively correlated, but squared GDP and SRH are positively correlated.

### Heterogeneity analysis

The effect of CHCs on SRH may vary according to individual differences. To address these different traits, this study divides the entire sample into several subgroups in terms of gender, new or old generations of migrants, educational background, and monthly income. For generations of migrants, according to the classification criteria for research on the new generation of migrant workers in China, those born before 1980 are defined as the old generation of migrants, and those born after that are defined as the new generation of migrants. For monthly income, this study categorized the sample regarding the overall mean as well as the sample distribution in each group. Education level was also classified according to this method.

Table 5 shows the results of the heterogeneity analysis based on gender and generation of migrants. According to the results, the effect of CHCs on female SRH was similar to the effect in the total sample, while the effect in the male group was proven to be less. Women's health is more vulnerable, so having close access to health services will greatly benefit their health status. Compared to Models 7 and 8, the effect of CHCs on SRH is higher in the new generation of migrants. Table 6 shows that CHCs have a greater effect on migrants with higher education levels. Well-educated migrants are more willing to access health resources (41). Meanwhile, CHCs have heterogeneity in the income of migrants; that is, they have a greater effect on the health promotion of individuals with higher income.

**Table 5:** Effect of CHCs on SRH by gender and age.

**Variables**	**Model 5**	**Model 6**	**Model 7**	**Model 8**
	**Female**	**Male**	**New generation of migrants**	**Old generation of migrants**
CHC	0.102***	0.083***	0.109***	0.072***
	(1.108)	(1.086)	(1.115)	(1.075)
Control variables	YES	YES	YES	YES
Provincial dummy variables	YES	YES	YES	YES
*N*	62,210	65,477	72,066	55,621
chi2	5,789.356	5,185.408	2,250.260	4,878.204
ll	−25,771.223	−26,443.242	−23,146.694	−28913.138

***represent significance at the 1% levels, Exp (B) display in parentheses.

**Table 6:** Effect of CHCs on SRH by education and monthly income.

**Variables**	**Model 9**	**Model 10**	**Model 11**	**Model 12**
	**Primary school or below**	**Above primary school**	**Monthly income ≤ 3500 RMB**	**Monthly income > 3500 RMB**
CHC	0.062*	0.095***	0.059***	0.149***
	(1.064)	(1.100)	(1.061)	(1.160)
Control variables	YES	YES	YES	YES
Provincial dummy variables	YES	YES	YES	YES
*N*	19,313	108,374	83,335	44,352
chi2	2,036.536	7,248.084	7,524.404	2,969.043
ll	−10,603.437	−41,20.698	−36,669.957	−15,461.360

*** and *represent significance at the 1 and 10% levels respectively. Exp (B) display in parentheses.

### Explanatory mechanism

The results of the empirical analysis above show that CHCs have significantly positive effects on SRH. This section explains the mechanism through which CHCs impact migrants' SRH. For this purpose, questions related to SRH were chosen to further investigate this mechanism.

Tables 7, 8 report the regression results after adding both explanatory and control variables. Models 13, 15, and 16 show that CHCs and migrants enhancing their awareness of health-related policies and knowledge are statistically significantly correlated, indicating that having CHCs in the community can help migrants receive health education and increase their health knowledge like the “National Basic Public Health Service Project,” as well as improve policy accessibility. In turn, this will make migrants pay more attention to their health and improve their health status. Models 14 and Models 17–20 show that CHCs also correlate with migrants' health-related behaviors by offering accessible health services. CHCs were significantly positively correlated with migrants establishing health records, going to CHCs when sick, and taking physical examinations. In addition, CHCs are significantly negatively correlated with the time from residence to medical service, which means that CHCs will help migrants go for medical treatment once they are needed. These health-related behaviors contribute to the early detection and treatment of diseases among migrants, thereby improving their health status. Thus, Hypothesis 2 was verified.

**Table 7:** Explanatory mechanisms of CHC on SRH at the destination (1).

**Variables**	**Model 13**	**Model 14**	**Model 15**	**Model 16**
	**Whether heard of the “National Basic Public Health Service Project”**	**Whether establish a health record**	**Whether understand the national public health policy**	**Whether received health education**
CHC	0.053***	0.085***	0.053***	0.046***
	(1.054)	(1.089)	(1.054)	(1.047)
Control variables	YES	YES	YES	YES
Provincial dummy variables	YES	YES	YES	YES
*N*	127,687	97,018	127,687	116,228
chi2	7,277.301	9,359.219	7,277.301	9,032.109
ll	−82,385.101	−57,088.638	−82,385.101	−63,922.575

***represent significance at the 1% levels, Exp (B) display in parentheses.

**Table 8:** Explanatory mechanisms of CHC on SRH at the destination (2).

**Variables**	**Model 17**	**Model 18**	**Model 19**	**Model 20**
	**Time from residence to medical service**	**Whether a specific disease is treated promptly on time**	**First consultation in CHC**	**Whether received follow-up assessment/physical examination at CHC**
CHC	−0.075***	−0.013	0.288***	0.186***
	(0.003)	(0.987)	(1.334)	(1.204)
Control variables	YES	YES	YES	YES
Provincial dummy variables	YES	YES	YES	YES
*N*	127,687	77,762	63,502	6,712
*R*2	0.025			
chi2		2,308.713	1,865.022	412.463
ll	−71,610.837	−50,993.036	−30,083.121	−4,071.331

***represent significance at the 1% levels, standard error displays in parentheses in Model 17, Exp (B) display in parentheses.

### Robustness test

For the robustness test, this study used sickness or being unwell as an independent variable to construct another regression model, using the survey question “Have you been sick or unwell in the last year? (And listed several common diseases)” and “yes” coded as “1”, “no” coded as “0”. The results showed that having CHCs in the community was significantly negatively correlated with migrant illness. To be more specific, Table 9 shows that after controlling for other variables, migrants living in a community with CHC were less likely to get unwell, with an odds ratio of 0.925, than those without CHCs. This result verifies our hypothesis 1 once again.

**Table 9:** Robustness test regression results (1).

**Variables**	**Model 21**	**Model 22**	**Model 23**	**Model 24**
Sickness or not	−0.082***	−0.081***	−0.082***	−0.078***
	(0.922)	(0.922)	(0.921)	(0.925)
Control variables	YES	YES	YES	YES
Provincial dummy variables	YES	YES	YES	YES
*N*	127,687	127,687	127,687	127,687
pseudo *R*-sq	0.034	0.035	0.036	0.037
ll	−85,461.6	−85,446.8	−85,351.2	−85,247.2

***represent significance at the 1% levels, Exp (B) display in parentheses.

Four health options were set in the questionnaire and binary variables were used in the benchmark regression. In order to check the robustness of the results, we used the setting of “1–4” to conduct the regression again. Table 10 shows that CHCs were still positively correlated with SRH, at a significance level of 0.01. Therefore, the benchmark regression results are robust.

**Table 10:** Robustness test regression results (2).

**Variables**	**Model 25**	**Model 26**	**Model 27**	**Model 28**
CHC	0.015***	0.015***	0.014***	0.013***
	(0.003)	(0.003)	(0.003)	(0.003)
Individual characteristics	Y	Y	Y	Y
Employment characteristics		Y	Y	Y
Family characteristics			Y	Y
Region characteristics				Y
Provincial dummy variables	Y	Y	Y	Y
*N*	127,687	127,687	127,687	127,687
adj. *R*-sq	0.115	0.127	0.132	0.133

***represent significance at the 1% levels, Exp (B) display in parentheses.

This study also uses the propensity score matching (PSM) method to test the robustness of Model 1–4 to further verify the impact of CHCs on the health of the migrants. PSM was used to match and maintain commonly supported samples. The PSM method found samples similar to the treatment group (living in a community with CHC) from the control group (living in a community without CHC) through the counterfactual analysis framework to construct the counterfactual state of the treatment group corresponding to the control group samples. Finally, the average treatment effect on the effect of CHCs on health was obtained using the logit model for analysis. Table 11 shows the PSM logit results.

**Table 11:** Results of PSM-logit.

**Variables**	**Model 29**		**Model 30**		**Model 31**		**Model 32**	
	**B**	**Exp (B)**	**B**	**Exp (B)**	**B**	**Exp (B)**	**B**	**Exp (B)**
CHC	0.103***	1.108	0.105***	1.110	0.099***	1.104	0.090***	1.095
Age	−0.059***	0.943	−0.055***	0.946	−0.054***	0.947	−0.055***	0.947
**Gender (female)**								
Male	0.242***	1.274	0.142***	1.152	0.145***	1.156	0.145***	1.156
**Nationality (others)**								
Han nationality	0.157***	1.170	0.144***	1.155	0.130***	1.139	0.126***	1.135
Year of education	0.036***	1.037	0.037***	1.038	0.027***	1.027	0.027***	1.027
**Marital status (unmarried)**								
Married	0.103***	1.108	0.097***	1.101	0.146***	1.157	0.148***	1.160
Migration Time	−0.012***	0.988	−0.014***	0.986	−0.013***	0.987	−0.013***	0.987
**Employment status (unemployed)**								
Employed			0.478***	1.613	0.437***	1.548	0.435***	1.544
Proportion of people aged 0–6 in the family					0.006	1.006	0.015	1.015
Proportion of people aged 65 and over in the family					−0.170***	0.844	−0.171***	0.843
Log of per capita household income					0.212***	1.236	0.215***	1.240
Unemployment rate							−7.002***	0.001
Per capita GDP^a^							−2.331***	0.098
Square of per capita GDP							0.100***	1.105
*N*	127,685	127,685	127,685	127,685	127,685	127,685	127,685	127,685
pseudo *R*-sq	0.101	0.101	0.105	0.105	0.107	0.107	0.108	0.108

^a^Both of the per capita GDP and square of per capita GDP have been taken logarithm.

***, **, and *represent significance at the 1, 5, and 10% levels, respectively.

Furthermore, there was a gap between migrants' subjective health cognition (SRH) and their objective health performance (sickness or not). This study investigated the impact of CHCs on the SRH of migrants. Therefore, further studies are needed to determine the cause of this gap and the underlying mechanism.

## Discussion

### Main findings

The main purpose of this study was to investigate the potential impact of CHCs on the SRH of migrants in China, and to identify the factors associated with such an impact. We found that CHCs were significantly correlated with SRH among migrants. Our further tests showed that CHCs can increase migrants' awareness of basic health knowledge through multiple programs such as “National Basic Public Health Service Projects,” thus promoting their participation in health education lectures, so that they will seek medical attention promptly and finally get better health. Thus, one potential mechanism that could explain the result is that CHCs can help migrants become aware of health knowledge and thereby prevent the occurrence of diseases, which is in line with the “Anderson model”. Previous studies have reported similar results. For instance, in one study, 38 participants were assessed before and after a one-hour CHC health education course about breast cancer health, and findings showed that the incidence of self-examination and scores on the accuracy of breast self-examination practice were significantly increased immediately following the intervention (42). Similar results have been found in studies of high-risk smokers (43) and low-income Chinese-American prenatal patients (44). Although the subjects of these studies were different, what they have in common is that they all lack information about some aspect of health. Our study reaffirmed that for migrants with little health knowledge, health education provided by CHCs will help improve their health.

Another possible mechanism is that CHCs can improve migrants' health behaviors. CHCs improve health by offering medical services in regions where healthcare resources are limited (45). There is also evidence that CHCs can promote health behaviors. For instance, a study of a CHC in West Los Angeles showed that the most vulnerable homeless individuals with the highest health risk were willing to receive CHC services, return for follow-up visits, and utilize services at least as much as low-income domiciled patients (37). Therefore, for migrants who are restricted by institutions and cannot enjoy more medical resources, the medical services provided by CHCs can undoubtedly help them maintain their health (46). The higher levels of SRH in people living in a community with CHC in our sample may be related to both of these mechanisms.

In addition, we found that not all impacts of CHCs behaved similarly across groups, and people with different characteristics had an inconsistent effect on SRH. More specifically, those who were male, younger, received more education, and with more stable employment reported better SRH. Males have better SRH in general and are less likely to be influenced by CHCs, which is consistent with previous studies suggesting that females are less confident about their health and are more sensitive to health problems (47). Younger people had better SRH, which is consistent with prior research (48), which could be because young people are not prone to serious diseases (49), and convenience is their primary concern when there is a real need for medical treatment (50). Thus, CHCs are convenient choices to meet the needs of younger migrants. Our research is also consistent with previous studies showing that low education is strongly associated with self-rated poor health and less utilization of public health services (51, 52). The results also revealed that the stability of migrants has an impact on their health; that is, the more stable the employment, the better the SRH. This finding is in line with previous studies showing that relatively more stable migrants have a greater opportunity to enroll in medical insurance, resulting in improved health (53). The result of the correlation between per capita household monthly income and SRH is in line with earlier studies on the correlation between income and health that there is a causal relationship between low-income and poor health (54).

Our findings are in contrast with those of an earlier study on the impact of the number of elderly (> 65 years) people in the family on SRH. Our findings suggest that having fewer elderly family members may result in better SRH (55). However, a prior study in South India did not find a significant correlation between the number of family members and SRH. Our discrepant findings may stem from the fact that our survey was conducted in countries with different cultural backgrounds.

### Strengths and limitations

The main contributions of this study are as follows: First, the “epidemiological paradox” only describe the health gap between migrants and non-migrants, as well as the health attrition of migrants, and does not provide a solution to this problem. By testing the mechanism by which CHCs improve migrants' health, this study provides a possible way to solve this problem. Second, a better understanding of the factors associated with migrant health can assist in the development of programs that improve the quality of life of one of the most vulnerable groups in China. Third, this study uses a large national sample of data to empirically examine the effect of CHCs on improving migrant health. This is an important addition to the current literature on migrant health.

Further research is needed to explore how CHCs affect migrants' psychological health. We know that CHCs improve migrants' SRH; however, it is unknown whether this pattern is similar to that of psychological health. The CMDS questionnaire was designed by the National Health Commission and the original questionnaire did not include mental health statements or more specific health rating options. Therefore, we recommend another follow-up study in the future that may use a better-designed scale to extend the investigation on this topic. Meanwhile, we use information about whether the respondents' made use of CHCs services in the mechanism test, but since the original questionnaire did not cover use barriers, follow-up studies could be supplemented in this aspect.

The study has some limitations. First, although a longitudinal study using earlier data would make the findings more robust, and the CMDS data have been collected for many years, the questions of the annual questionnaire are not consistent, except for information on basic demographic and employment characteristics. Currently, only data from 2017 include information about CHCs; hence, no longitudinal analysis can be performed. Second, the results of this study may not be precise enough because of the use of 0–1 SRH. Therefore, we suggest that future research should use a more fully designed scale, including psychological problem and illness statements, and refine the options to further explore the findings of this study.

## Conclusion

The confluence of two of the largest demographic changes of the twenty-first century in China, which are population aging and migrant growth, demands our attention. An important concern that we are facing is what can be done to stop the “epidemiological paradox” to ensure that migrants return to their hometown for retirement life with better health. By identifying factors that contribute to the deterioration of health among migrants, we come closer to being able to identify programmatic approaches to address this critical question. For instance, the “Anderson model” and the current study have shown that enrolling in medical insurance can improve migrant health. However, given that institutional constraints still exist for migrants participating in urban employee medical insurance, we need to see if there are other factors that play the same role. By using a large, nationally representative sample of migrants in China, measure of SRH, and a comprehensive set of correlates known to be associated with SRH, we offer a cautious conclusion that by offering health knowledge that migrants lack and provide convenient health services, CHCs can improve the SRH of migrants in China. Therefore, in the context of the reality that institutional restrictions cannot be completely lifted for the time being, providing support for medical institutions that can be used by migrants will help improve their health.

## Data availability statement

Publicly available datasets were analyzed in this study. This data can be found here: China Migrants Dynamic Survey https://chinaldrk.org.cn/wjw/#/home.

## Author contributions

A-LM took leadership and responsibility for the whole research and write the final manuscript. Y-NL worked on the statistical analysis of the data and improved the model design. Y-KT responsible for preliminary data analysis. All authors read and approved the final manuscript. All authors contributed to the article and approved the submitted version.

## Funding

This article was funded by the National Social Science Fund of China (grant no. 20CGL040) and Humanities and Social Science Research Project of Ministry of Education (grant no. 21YJC790068).

## Conflict of interest

The authors declare that the research was conducted in the absence of any commercial or financial relationships that could be construed as a potential conflict of interest.

## Publisher's note

All claims expressed in this article are solely those of the authors and do not necessarily represent those of their affiliated organizations, or those of the publisher, the editors and the reviewers. Any product that may be evaluated in this article, or claim that may be made by its manufacturer, is not guaranteed or endorsed by the publisher.

## Abbreviations

CHC: community health center
CMDS: China Migrants Dynamic Survey
HIE: healthy immigrant effect
NCMS: new cooperative medical scheme
PSM: propensity scorematching
SRH: self-rated health
UEBMI: urban employee basic medical insurance
URBMI: urban resident basic medical insurance
PPS: proportional to population size
RMB: renminbi
GDP: gross domestic product
